# An Odor Trace Visualization System Using a Two-Dimensional Backside Scattering Localized Surface Plasmon Resonance Gas Sensor

**DOI:** 10.3390/s23239525

**Published:** 2023-11-30

**Authors:** Zhongyuan Yang, Fumihiro Sassa, Kenshi Hayashi

**Affiliations:** Graduate School and Faculty of Information Science and Electrical Engineering, Kyushu University, 744 Motooka, Nishi-ku, Fukuoka 819-0395, Japan; yang.zhongyuan.327@s.kyushu-u.ac.jp

**Keywords:** gas sensor, odor trace visualization, localized surface plasmon resonance, spatial distribution, chemical imaging

## Abstract

Odor information fills every corner of our lives yet obtaining its spatiotemporal distribution is a difficult challenge. Localized surface plasmon resonance has shown good sensitivity and a high response/recovery speed in odor sensing and converts chemical information such as odor information into optical information, which can be captured by charge-coupled device cameras. This suggests that the utilization of localized surface plasmon resonance has great potential in two-dimensional odor trace visualization. In this study, we developed a two-dimensional imaging system based on backside scattering from a localized surface plasmon resonance substrate to visualize odor traces, providing an intuitive representation of the spatiotemporal distribution of odor, and evaluated the performance of the system. In comparative experiments, we observed distinct differences between odor traces and disturbances caused by environmental factors in differential images. In addition, we noted changes in intensity at positions corresponding to the odor traces. Furthermore, for indoor experiments, we developed a method of finding the optimal capture time by comparing changes in differential images relative to the shape of the original odor trace. This method is expected to assist in the collection of spatial information of unknown odor traces in future research.

## 1. Introduction

The human olfactory system detects various gas molecules in the air, leading us to perceive different odors [1,2]. Generally, what we refer to as ‘smelling’ is actually the detection of specific gaseous substances associated with an odor. Gas sensors play an indispensable role in the field of odor/gas detection [3,4,5,6]. In residential buildings, commercial facilities, and factories, gas alarm systems detect gas leaks or harmful gases (e.g., carbon monoxide, natural gas, and hydrogen sulfide) to ensure the safety of people [7]. In chemical and pharmaceutical plants, concentrations of raw material gases or intermediate products are monitored to ensure product quality and production efficiency [8]. In cell culture processes, gases such as oxygen and carbon dioxide are identified and their concentrations monitored to maintain the required gas environments [9]. In addition, researchers are expanding the capabilities of existing gas sensors to more efficiently acquire and use odor information.

The development of methods for detecting types of gases that are difficult to separate conventionally, using special adsorption materials, has become a hot topic of research in recent years [10]. In particular, the use of materials with microstructures or highly designed chemical properties has improved the ability to selectively adsorb and separate specific gases. Changes in the vibration frequency of the quartz crystal microbalance can be measured when gas molecules are adsorbed inside metal–organic frameworks [11]. Changes in the threshold voltage or conductivity of a field-effect transistor can be measured when gas molecules are adsorbed on the surfaces of carbon nanotubes [12]. Changes in the electrical resistances of materials can be measured when gas molecules are adsorbed on the surfaces of metal oxides [13]. Shinohara et al. reported a new molecularly imprinted filtering adsorbent, which comprises an adsorbent base layered with a molecularly imprinted polymer, to enhance the capability for odor molecule recognition [14]. In addition, there is pertinent research on determining types of volatile organic compounds using an array of multiple gas sensors and information processing. Lonergan’s team reported a gas sensor array for which individual sensor properties are tuned by varying the carbon black content [15] and Ge et al. reported a fully inkjet-printed gas sensor matrix with gas-selective materials [16].

Researchers have long investigated the selectivity and intensity of odor molecules, but describing the spatiotemporal distribution of these odors is a complex task [17,18,19,20]. Odor plumes and odor traces provide information on the odor’s spatiotemporal distribution but have differences. An odor plume describes the pattern or shape of the diffusion of an odor from its source. It spreads out like a ‘feather’, sometimes presenting in a fan shape or irregular form. The shape and intensity of the plume are affected by various factors, including wind speed, wind direction, temperature, and humidity. Understanding odor plumes is crucial in environmental science and ecology, as they help researchers identify and track specific odor traces, such as those of pollutants [21,22,23]. Meanwhile, an odor trace usually refers to the trail or imprint of an odor left by a particular source on a specific object’s surface. Odor traces are somewhat fixed to their source and fade over time. This concept is especially important in crime scene investigations, search and rescue operations, and animal behavior studies, where tracking dogs might be used to trace the odor of a person or object [24,25,26]. Specifically, the distribution information (shape) of an odor trace often intuitively reveals the source that left the trace.

Sensing in both the spatial and temporal domains of odor traces is complicated. Researchers attempt to obtain them by using different kinds of gas sensors [17]. The most typical example is the MOX gas sensor, which offers sub-ppm sensitivity, cost-effectiveness, and is easy to fabricate [27]. Using mobile robots equipped with gas sensors to locate odor sources through specific tracking methods is a classic example of utilizing the spatial distribution information (concentration distribution) of an odor plume [28]. However, two-dimensional sensing with MOX sensors relies on an array of multiple sensors, making them unsuitable for direct two-dimensional sensing. Additionally, it cannot directly convert sensitivity signals into visual signals for visualization, and the longer recovery time (several tens of seconds) makes real-time detection difficult. Biosensors can be easily unfolded in a two-dimensional plane, and visualization can be achieved through changes in fluorescence on the sensor due to interactions with target substances. Research groups have recently reported the acquisition of the spatial distributions of gases using a camera-based imaging system using a gas-responsive material spread out on a plane (i.e., a two-dimensional (2D) gas imaging system). Yoshioka et al. devised an odor imaging system based on multispectral fluorescence imaging, which visualized and discriminated components for a hand-shaped odor mark and airflow containing different odorants [29]. Itani et al. developed a gas-imaging system using alcohol dehydrogenase for imaging the concentration distribution of acetaldehyde in breath and transdermal vapor [30]. These kinds of biosensors have high sensitivity at the ppb level, but they are limited to detecting substances that can introduce a fluorescent response. Additionally, their response and recovery speeds limit the sensors’ ability to capture real-time changes in odor traces.

Localized surface plasmon resonance (LSPR) has shown great potential in the field of 2D odor trace visualization. LSPR is a phenomenon observed in nanomaterials, such as nanoparticles and nanorods, where electrons collectively oscillate in response to incident light [31]. This resonance enhances the absorption of light at specific wavelengths. LSPR is highly sensitive to changes in the surrounding medium, such as the introduction of gas molecules near the nanoparticles. LSPR is widely used in chemical sensors and biosensors based on optical signals [32,33,34]. Controllable gold nano seeds are formed by annealing a glass substrate sputtered with gold nanoparticles [35,36]. Through light exposure, the growth of silver nanoparticles is induced and controlled to form an Au/Ag core-shell structure. The growth time is adjusted to control the particle size and inter-particle distance and thus achieve the desired structure [37,38]. The fabrication method demonstrates that LSPR sensors can be easily fabricated as two-dimensional sensors and, owing to their ability to change optical characteristics in response to surrounding substances, facilitate effective visualization. In our previous research, we confirmed that LSPR gas sensors have sensitivity at the ppm level [39]. Moreover, these sensors exhibit a very high response/recovery speed. Targeting volatile substances adsorbed on the ground, our team has developed an odor robot equipped with a transmission-type LSPR gas sensor that excels in terms of its response speed to a rapidly switching gas environment every 20 ms. The robot accurately identifies the location of odor sources while moving at a high speed of 10 cm/s [40].

We are presently conducting research to visualize the mixture of volatile substances on the ground using, for example, the one-dimensional robot system mentioned above and obtaining gas trace information from 2D information. We have successfully visualized ethanol gas flow (using a transmissive type of detector) [41] and odor traces (using a reflective type of detector) [42] using a 2D-LSPR substrate on a fixed experimental platform. However, the transmissive type of detector cannot detect odor traces on the ground, and the reflective type of detector captures the camera lens in the image and the strong reflection of its substrate interferes with subsequent image processing.

The present study developed a new kind of odor trace visualization system based on an Au/Ag LSPR substrate for visualization of on-ground odor traces to obtain its spatiotemporal change. We conducted a comparative experiment to ensure the reliability of the visualization images obtained by the system under environmental effects. Furthermore, by observing changes in the visualized images of the odor trace over time relative to the original shape of the odor trace, we determined the optimal time for capturing and detecting unknown odor traces.

## 2. Materials and Methods

### 2.1. Visualization Concept and Principle of the On-Ground Odor Trace Imaging System

**Figure 1:**
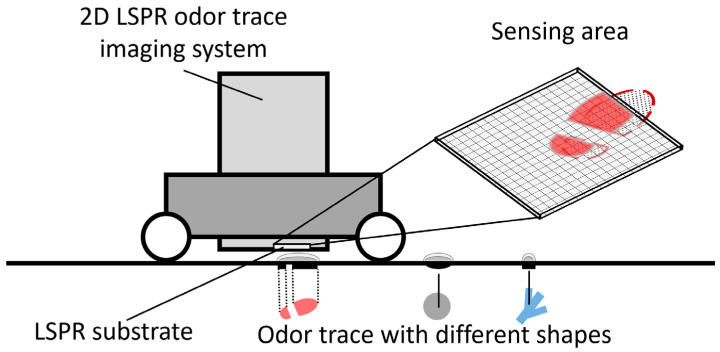
Concept of collecting odor trace images (spatial information from an on-ground odor trace) with the two-dimensional (2D) localized surface plasmon resonance (LSPR) odor trace imaging system.

Figure 1 presents the concept of the on-ground odor trace imaging system that visualizes the otherwise invisible world of on-ground odor traces. These odor traces, while not discernible to the naked eye, emit volatile organic compounds and the odor trace itself has a specific spatial distribution in two-dimensional (shape). The imaging system comprises a charge-coupled device (CCD) camera, LSPR sensor substrate, and light-emitting diode (LED) light source. The core of the system is the LSPR substrate, which has a high response speed to volatile organic compounds and converts the chemical information of volatile organic compounds into optical information. The LSPR substrate consists of noble metal nanoparticles, such as Au and Ag, arranged on a glass substrate. The optical properties of the substrate are affected by volatile organic compounds from odor traces approaching the nanoparticles on the substrate. Variance in properties across the substrate presents a visual pattern, which is captured by a CCD camera. The visualized image of emitted gas components from the odor trace closely aligns with the actual shape of the invisible odor trace, given that the substrate is placed mere millimeters from the odor trace. In essence, the system transforms the chemical information of the odor trace into tangible optical images, bridging the unseen chemical domain to our visual world.

**Figure 2:**
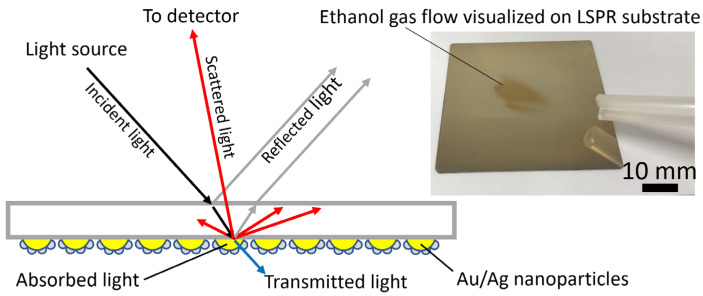
Visualization principle of the odor trace imaging system with an Au/Ag core-shell LSPR substrate that is based on changes in scattering and absorption captured by a charge-coupled device (CCD) camera when gas molecules approach the Au/Ag nanoparticles.

The visualization principle is shown in Figure 2. The optical property of the substrate is determined by the absorption and scattering properties of the Au/Ag core-shell nanostructure on the surface of the substrate. These properties relate to the size, shape, and dielectric environment of the nanostructure. When gas molecules approach the nanostructure, they affect the surrounding dielectric environment and/or the chemical nature of the surface of the nanostructure. The light scattered from the LSPR sensor surface, which is changed by the dielectric change of the surrounding media, can be observed and visualized using a CCD camera from the backside. The intensity of scattered light (*S*) is calculated as:

*S* = *I* − *R* − *T* − *A*, (1)

where *I* is the intensity of incident light, *R* is the intensity of reflected light, *T* is the intensity of transmitted light, and *A* is the intensity of absorbed light.

The trace of saturated ethanol gas blown onto the LSPR substrate is shown in the right part of Figure 2. The chemical information of an approaching ethanol gas flow is converted into changes in color and brightness (optical information) by the substrate and ultimately captured by the CCD camera. At the same time, as the substrate has a high response/recovery speed, it immediately returns to its original state after the ethanol gas disappears, leading to a response/recovery in the surrounding dielectric environment, such that the substrate can effectively adapt to and record a rapidly changing gas environment.

### 2.2. Fabrication of an LSPR Sensor Substrate with an Au/Ag Core-Shell Nanostructure

Silver nitrate and trisodium citrate were purchased from Wako, Japan.

Au nano seeds were fabricated on a glass substrate with dimensions of 50 mm × 50 mm × 1 mm. The glass substrate was washed with pure water, acetone, and ethanol in an ultrasonic cleaner separately and dried in a nitrogen gas steam. The glass substrate was then put into a sputter machine (SC-701HMC II, Sanyu Denshi, Tokyo, Japan) for Au nanoparticle deposition by sputtering (20 mA, 8 s). An annealing treatment was carried out in a programmable electric furnace (SMF-1, As One, Osaka, Japan) at 580 °C for 8 h to produce an Au nano-island structure on the glass substrate.

Solutions of silver nitrate (10 mM, 5 mL), trisodium citrate (100 mM, 4 mL), and pure water (11 mL) were mixed together before light exposure. The substrate with the Au nano-island structure was immersed in the mixed solution and exposed to light from a halogen light source (MHF-150L, Moritex, Kanagawa, Japan) to provide the power of 2.0 mW/cm^2^ to the Au/Ag core-shell nanostructure. The substrate was rinsed with pure water and dried in a steam of nitrogen gas.

### 2.3. Fabrication of a 2D LSPR Odor Trace Imaging System

**Figure 3:**
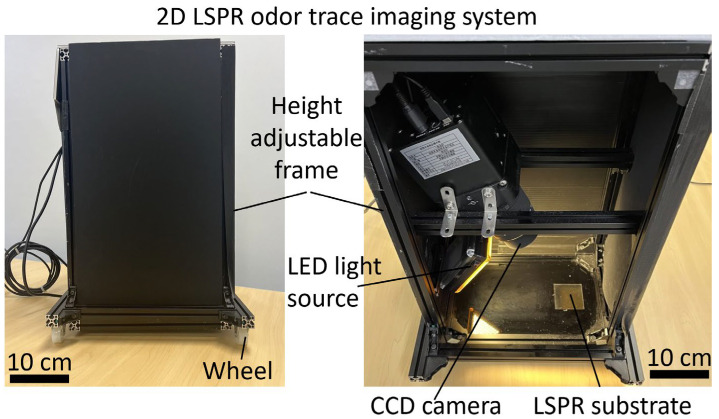
Overview of the 2D LSPR odor trace imaging system (**left**) and photograph of the imaging system (**right**), comprising a CCD camera, light-emitting diode (LED) light source, and LSPR sensor substrate.

A photograph of the 2D LSPR odor trace imaging system is shown in Figure 3.

The frame of the imaging system is made of aluminum alloy and has dimensions of 30 cm × 20 cm × 50 cm. The height of the overall device is adjusted to ensure that the measuring area of the LSPR substrate remains close to the odor trace in different experimental environments. The overall sensor system is covered with black cardboard to block out light. A CCD camera (ML4710-1-BB, FLI, New York, NY, USA) is set at the top of the sensor chamber to obtain the intensity change on the sensor substrate. A square hole is cut in the chamber floor 15 cm below the camera for the LSPR sensor substrate. An LED light source (Ulanzi 96 LED Video Light, Ulanzi, Shenzhen, China) is set at the left of the chamber, with the angle between the light source and substrate being approximately 30 degrees to avoid direct reflection. The wavelength range of the LED light source is 400 nm to 800 nm with its peak at 600 nm. An ice pack is placed next to the substrate to suppress the desorption of gas molecules on the substrate surface due to the internal temperature of the chamber being higher than the external temperature, a condition caused by heat dissipation from the camera.

## 3. Results and Discussion

### 3.1. Characterization of the Au/Ag Core-Shell LSPR Substrate

**Figure 4:**
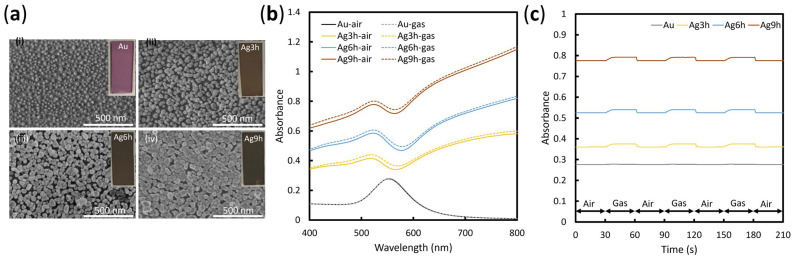
Characteristics of the LSPR sensor substrate. (**a**) Scanning electron microscope image of the Au/Ag core-shell nanostructure. (i) Au nanoparticles only. (ii)–(iv) Au nanoparticle layers with growth at different times. (Insets) Photographs of the LSPR sensor substrates (**b**) Absorption spectra of the LSPR substrates exposed to air and ethanol gas. (**c**) Absorbance changes when the LSPR substrates were exposed to air and ethanol gas.

Figure 4a presents scanning electron microscope images and photographs of the Au nanoparticle substrate and substrates with Ag nanoparticle growth for 3, 6, and 9 h, showing the Au/Ag core-shell nanostructure. As the Ag grows, the color of the substrate gradually darkens, changing from pink to dark brown. The scanning electron microscopy of the Au nanoparticle substrate shows a dispersed island structure, with particle sizes ranging from 10–30 nm. With the growth of Ag, the diameter of the nano-islands increases to approximately 50 nm and the distance between the islands decreases, such that the structure becomes denser.

The absorption spectra of each substrate in air and saturated ethanol gas are shown in Figure 4b. A solid line shows the absorption spectrum when the substrate was exposed to air. A broken line shows the absorption spectrum when the substrate was exposed to ethanol gas. As the Ag growth time increases, the absorption of visible light by the substrate increases and the intensity of transmitted light correspondingly decreases. This confirms that the substrate effectively reduces the effect of transmitted light from outside on imaging when used in the 2D imaging system.

The substrates were exposed to an environment that switched between air and ethanol gas every 30 s to investigate the effect of Ag nanoparticle growth on sensitivity. Figure 4c presents the absorption changes of each substrate at a wavelength of 550 nm. From bottom to top are the results for the Au nanoparticle substrate and the substrates with 3, 6, and 9 h of Ag growth. The changes in the absorption value are 0.002, 0.015, 0.015, and 0.016, respectively. It is observed that the growth of Ag enhanced the sensitivity of the substrate, while its absorbance change remained stable. The red-shifted shoulder in the absorbance spectrum (Figure 4b) is due to a resonance generated by coupling between the nanoparticles [43]. Therefore, the growth of silver effectively reduces the noise generated by transmitted light without affecting the sensitivity.

In our previous study, we verified that the optical properties of LSPR substrates can be controlled by adjusting the amount of light exposure (growth time) [44]. Although it is not possible to create exactly identical LSPR substrates, those obtained with the same exposure time exhibit similar optical characteristics. The susceptibility of the Ag shell to oxidation presents a challenge for future research, as the reverse growth of controlled Au on Ag seeds is not achievable in this fabrication process. To minimize the influence of Ag’s susceptibility to oxidation on the experimental results, all data were collected within one week of substrate preparation.

### 3.2. Comparative Experiment of the Visualization of an A-Shaped Odor Trace

As the optical properties of the LSPR substrate do not simply represent its performance in the odor trace imaging system, we conducted a comparative experiment for further analysis and evaluation. The LSPR substrate is greatly affected by factors such as temperature, humidity, and ambient light, and the experiment is thus designed to accurately obtain the differences in imaging results between the LSPR substrate and non-LSPR substrate for the same odor trace under the same conditions.

**Figure 5:**
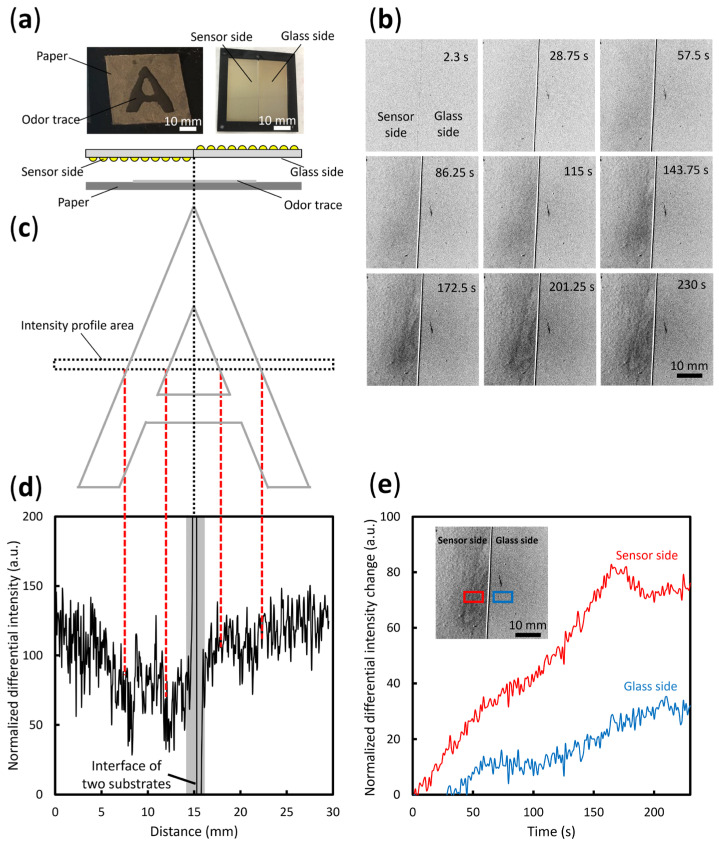
Comparative experiment of odor trace visualization. (**a**) Experiment set up. (**b**) Differential images of the A-shaped odor trace over time. (**c**) Intensity profile area. (**d**) Intensity changes on the sensor side and glass side in the differential image at 230 s. (**e**) The average change in intensity between the sensor side and glass side over time. The inset shows the two sampling areas.

The odor trace visualization system shown in Figure 3 was placed in a dark room. Two glass substrates, each with dimensions of 25 mm × 50 mm, were fabricated with a 5-nm Au layer and an Ag layer grown for 9 h. The two substrates were placed on a poly(methyl methacrylate) (PMMA) frame. The substrate on the left was fabricated with the Au/Ag core-shell nanostructure (sensor side) facing downward, and the glass side of the substrate on the right (glass side) facing downward, for a comparative experiment. As shown in Figure 5a, black cardboard with dimensions of 5 cm × 5 cm was coated with a hydrophobic solvent (Fluoro Surf, Fluorotech, Kasugai, Japan) in areas outside of an “A” shape and dried for 10 min. The area within the “A” shape was coated with 95% ethanol solution to create an A-shaped odor trace immediately before image capture. The LSPR sensor substrate was positioned directly above the odor trace at a distance of approximately 4–5 mm. The ethanol gas concentration, measured by a PID sensor (Phocheck+, Ion Science, Fowlmere, UK) directly above the odor trace at a distance of 5 mm, was approximately 7000 ppm.

The exposure time of the CCD camera was set at 0.25 s and 200 images were taken with intervals of 0.9 s over a period of approximately 230 s to observe the intensity changes on the LSPR sensor substrate and glass substrate due to the A-shaped odor trace. After background subtraction using the first image as the reference image, differential images were obtained as shown in Figure 5b. Initially, within the first 57.5 s of capturing, there was little to no observable change on either the sensor side or the glass side. At approximately 115 s, there was a slight decrease in intensity on the sensor side. At approximately 140 s, the “A” shape of the odor trace began to appear from top to bottom on the sensor side, and by approximately 200 s, the complete “A” shape was visible. In contrast, there were hardly any notable changes in the images on the glass side in the capturing process. The overall change in intensity over time was clearer on the sensor side than on the glass side. It is speculated that due to the diffusion of ethanol gas, a concentration gradient formed from near to far, which affected the absorption and scattering properties of the substrate captured by the CCD camera.

To further investigate the difference between the sensor side and the glass side, the intensity profile for the area indicated in Figure 5c was plotted using ImageJ software (V 1.52a) with a line width of 20 pixels in Figure 5d. The line width of the profile is set in the longitudinal direction, and the average intensity over 20 pixels in the transverse direction is presented. The gray bar shows the position of the interface between the sensor side and the glass side. At approximately 3 and 7 mm to the left of the interface line on the sensor side, there are two clear valleys corresponding to the A-shaped odor trace. In contrast, at the corresponding positions on the glass side, there is hardly any change in intensity. This intensity profile on the one image effectively shows the difference in intensity changes due to the odor trace on the LSPR substrate and glass substrate on the same external environmental condition over time. The result confirms that the parts of the cardboard with and without the odor trace show differences on the substrate, creating a boundary. In the differential images, these differences produce a visible line that reveals the shape of the original odor trace.

Figure 5e presents the average differential intensity changes over time for the sensor side and glass side (where the inset shows the sampling areas). The sensor side shows a larger change in the normalized intensity of 80 over the course of approximately 160 s. The glass side shows a slight change in normalized intensity of 20 over the same period. At the location of the odor trace, owing to the diffusion of saturated ethanol gas that affects the optical properties of the substrate in the sampling area, the average intensity change increases over time. Meanwhile, the intensity change over time for the glass substrate serves as an effective reference for environmental effects.

In our previous research, we successfully utilized Au/Ag LSPR substrates to visualize various volatile gases including geraniol, pentadecane, piperitone, and eugenol [45]. In this experiment, we further achieved the acquisition of spatial distribution information (shape) of complex-shaped ethanol odor traces and their changes over time.

### 3.3. Indoor Environmental On-Ground Star-Shaped Odor Trace Visualization

**Figure 6:**
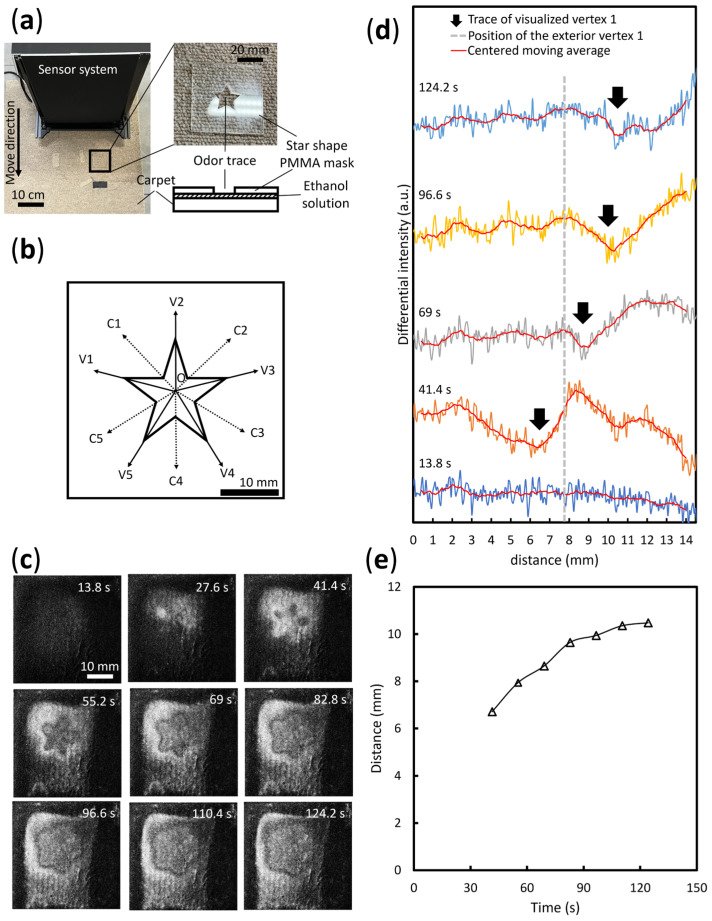
On-ground star-shaped odor trace visualization in an indoor environment. (**a**) Experimental setup. (**b**) Directions of intensity profiles in 10 directions. (**c**) Differential images of the star-shaped odor trace over time. (**d**) Intensity profile of the line V1 at different times. (**e**) Position shift of the visualized vertex 1 over time.

The sensor system was moved over a designed odor trace to obtain shape information in an indoor environment at room temperature, as shown in Figure 6a.

A black mark was made to indicate the stopping position of the imaging system after moving the system onto the carpet used in the experiment, ensuring that the LSPR sensor substrate was directly above the odor trace during the imaging process. A star-shaped hole was cut in a PMMA substrate with dimensions of 50 mm × 50 mm × 2 mm to be used as a mask. The overall size of the star opening was approximately 15 mm × 15 mm, the distance from the center to each of the five exterior vertices was 7.8 mm, and the distance from the center to each of the interior vertices was 3.9 mm. The mask was placed on the carpet, and the space between the carpet and the PMMA mask was wetted with ethanol solution to create a star-shaped ethanol odor trace, as shown on the right of Figure 6a. The distance between the odor trace and sensor substrate was adjusted to approximately 4–5 mm. The exposure time of the CCD camera was set at 0.5 s, and 100 images were taken with intervals of approximately 0.88 s over a period of approximately 138 s.

We marked the different directions in the original star shape to more conveniently describe the directions in the images. As shown in Figure 6b, starting from the left side, the five exterior vertices were sequentially labeled as exterior vertices 1 to 5 and the five interior vertices were labeled as interior vertices 1 to 5. Ten line arrows were drawn from the center of the star to the five exterior vertices and five interior vertices, marked as line arrows V1 to V5 and C1 to C5, to investigate the intensity change in different directions of the image. The length of each line was approximately 15 mm (i.e., 250 pixels in the image).

Differential images of the visualized star shape odor trace (with the background subtracted using the first image as the reference) are shown in Figure 6c. In the differential images, there is hardly any observable change for the first 13.8 s. At approximately 27.6 s, the overall brightness of the upper half of the image corresponding to the star-shaped odor trace starts to increase. At approximately 41.4 s, the upper three vertices of the star shape have formed distinct elliptical shadows in the image. At 55.2 s, the complete star shape is observed. The interior of the star shape is notably darker than the exterior, and there is a clear decrease in brightness at the edges of the star-shaped trace, forming a distinct star-shaped outline. From 69 to 124.2 s, the entire image continues to spread outward, gradually transforming into a pentagon and ultimately becoming an irregular shape. The dispersion of the odor is not symmetrical or uniform across the area. There is a faster rate of change in shape in the V1, V2, and V3 directions with the shape maintaining its initial form for a more extended period in the other directions. The defined edges of the star shape and the subsequent changes highlight the complexity of the odor diffusion process.

To more clearly show the changes in the image and the movement of the edge area of the odor trace during imaging, we plotted the intensity profile from the center O in the direction of exterior vertex 1, over a length of approximately 15 mm (250 pixels). The intensity changes along the arrow V1 at 13.8, 41.4, 69, 96.6, and 124.2 s are shown in Figure 6d. The line width was set at 20 pixels to reduce the effect of noise. Each arrow shows the position of visualized vertex 1. At 13.8 s, the intensity along the line arrow V1 does not change appreciably, which is consistent with the image displayed in Figure 6c. At 41.4 s, there is a notable valley at approximately 6.7 mm (as indicated by the black arrow in the image). Subsequently, at 69, 96.6, and 124.2 s, the valley gradually moves outward to 8.6, 9.9, and 10.5 mm, respectively. At 124.2 s, between 10.5 and 12.1 mm, there is a long valley with two intensity decreases in the graph. The movement of the valley suggests a dynamic diffusion of the odor emitted from the odor trace, likely expanding outward from its source.

For the differential images, the intensity profile is plotted within a length of 15 mm along the V1 direction. The position of the valley was recorded every 13.8 s from 41.4 to 124.2 s. These recorded valley positions represent the outline of the image of the odor trace visualized by the sensor substrate. Figure 6e shows the shift in the position of the valley corresponding to the start point of the edge of the visualized odor trace over time. From 41.4 to 82.8 s, the valley moves from 6.7 to 9.6 mm, indicating an appreciable outward movement over time. From 82.8 to 124.2 s, the valley moves from 9.6 to 10. 5 mm, corresponding to a smaller change in position. The speed of motion of the outline of the visualized odor trace in the differential images is approximately 0.07 mm/s from 41.4 to 82.8 s and 0.02 mm/s from 82.8 to 124.2 s.

**Figure 7:**
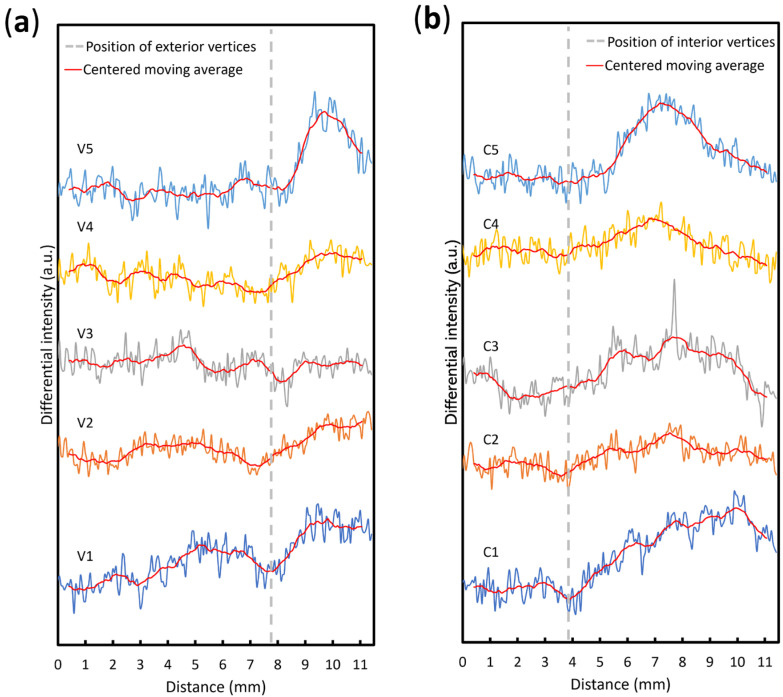
Intensity profiles in different directions. (**a**) Intensity profiles along V1 to V5 at 55.2 s. The grey broken line represents the positions of the five exterior vertices. (**b**) Intensity profiles along C1 to C5 at 55.2 s. The grey broken line represents the positions of the five interior vertices.

Figure 7a shows the change in intensity from the center of the star shape to each of the exterior vertices at 55.2 s. It is seen that at approximately 7.8 mm from the center, there is an appreciable intensity change, showing that the edge of the odor trace can be clearly observed in the image. Owing to uneven diffusion in different directions, the positions of the valleys are not the same, but they are all near the positions of the exterior vertices. Exterior vertices 1, 2, 4, and 5 have a greater intensity decrease around the valleys, making them clearer in the visual image. In contrast, V3 appears relatively blurred. Figure 7b shows a similar result at approximately 4 mm from the center for the interior vertices of the star.

The distance between the LSPR substrate and the odor trace affects image quality in various ways. In our experimental setup, the minimum achievable distance is 4–5 mm. Due to diffusion effects, a closer proximity of the substrate to the odor trace enhances the concentration difference in the local area of the substrate, resulting in clearer images. However, as the distance increases, the externally transmitted light passing through the substrate significantly impacts its visualization capabilities. Given the very fast response time of LSPR substrates (20 ms, as observed in our previous study [42]), we do not consider time costs to be a significant factor. We assume that the differential images captured at different times accurately represent the real-time distribution of the traces left on the substrate.

**Figure 8:**
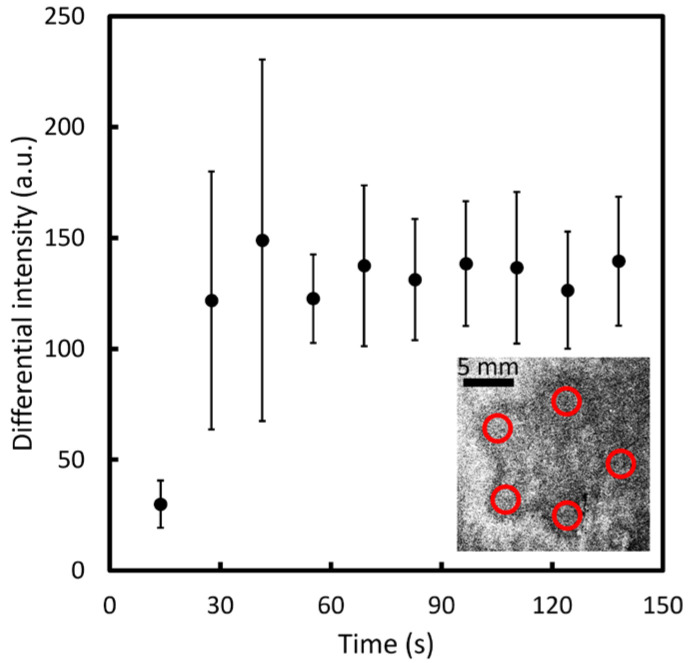
Intensity changes at the five vertices over a period of 138 s. Red circles represent the five sampling areas of the vertices in the visualized images.

In further investigating the temporal change in intensity, results were examined over areas having a diameter of approximately 1.2 mm (20 pixels) at each exterior vertex. Figure 8 shows that before 41.4 s, there is an appreciable increase in intensity in the differential images from 29.99 to 148.97 arbitrary units. The intensity in the areas becomes stable after 55.2 s, remaining between 122.65 and 139.52 arbitrary units. The results indicate that from around 55.2 s, the image obtained from the intensity changes due to the odor trace on the substrate provides relatively complete shape information about the odor trace. This method is considered to be an effective calibration method for determining the optimal observation time under various floor materials and experimental conditions.

## 4. Conclusions

In this study, we developed a 2D odor trace visualization system based on an LSPR substrate with an Au/Ag core-shell nanostructure, with the observation of light scattered from the backside.

The original images obtained from close-range (4–5 mm) odor traces on the ground, after subtraction analysis, show reasonably clear brightness changes due to the volatile gas ethanol diffused from the odor trace on the sensor substrate. The differential image provides an intuitive display of the spatial distribution information (shape) of invisible odor traces and their changes over time. Comparative experiments clearly demonstrated the ability of the LSPR substrate to capture an odor trace compared to a non-LSPR substrate under the same environmental conditions.

Indoor experiments demonstrated the system’s ability to capture an odor trace with a complex shape (star shape) set for real floor environments, such as a carpeted environment. In addition, we developed an experimental method to confirm the optimal observation time for the visualization image to match the original trace shape in real-world conditions. The optimal time for capturing a star-shaped odor trace was approximately 55 s after the imaging system stopped above the odor trace in an indoor experiment. This method can be used in future research, such as when equipping systems like robots, to obtain the spatial distribution information of the odor trace at a specific location.

## Data Availability

Data are contained within the article.
